# Preparation of Few-Layered MoS_2_ by One-Pot Hydrothermal Method for High Supercapacitor Performance

**DOI:** 10.3390/nano14110968

**Published:** 2024-06-02

**Authors:** Qingling Jia, Qi Wang, Lingshuai Meng, Yujie Zhao, Jing Xu, Meng Sun, Zijian Li, Han Li, Huiyu Chen, Yongxing Zhang

**Affiliations:** 1 Anhui Province Key Laboratory of Pollutant Sensitive Materials and Environmental Remediation, Anhui Province Key Laboratory of Intelligent Computing and Applications, Anhui Province Industrial Generic Technology Research Center for Alumics Materials, Huaibei Normal University, Huaibei 235000, Chinazyx07157@mail.ustc.edu.cn (Y.Z.); 2 School of Materials Science and Engineering, North University of China, Taiyuan 030051, China

**Keywords:** few layer, one-pot hydrothermal, MoS_2_, exfoliation, supercapacitor performance

## Abstract

Molybdenum disulfide (MoS_2_), a typical layered material, has important applications in various fields, such as optoelectronics, catalysis, electronic devices, sensors, and supercapacitors. Extensive research has been carried out on few-layered MoS_2_ in the field of electrochemistry due to its large specific surface area, abundant active sites and short electron transport path. However, the preparation of few-layered MoS_2_ is a significant challenge. This work presents a simple one-pot hydrothermal method for synthesizing few-layered MoS_2_. Furthermore, it investigates the exfoliation effect of different amounts of sodium borohydride (NaBH_4_) as a stripping agent on the layer number of MoS_2_. Na^+^ ions, as alkali metal ions, can intercalate between layers to achieve the purpose of exfoliating MoS_2_. Additionally, NaBH_4_ exhibits reducibility, which can effectively promote the formation of the metallic phase of MoS_2_. Few-layered MoS_2_, as an electrode for supercapacitor, possesses a wide potential window of 0.9 V, and a high specific capacitance of 150 F g^−1^ at 1 A g^−1^. This work provides a facile method to prepare few-layered two-dimensional materials for high electrochemical performance.

## 1. Introduction

In recent decades, it has been essential to develop a new generation of energy storage devices that are eco-friendly, sustainable, and capable of meeting the evolving energy demands [1,2,3]. Supercapacitors have attracted significant attention due to their superior power density and energy density, excellent cycle performance, and fast charge–discharge characteristics [4,5,6]. Supercapacitors can be divided into two categories (electrostatic double layer capacitors (EDLCs) and pseudocapacitors) based on the energy storage mechanisms [7]. Regarding EDLCs, the double-layer capacitor on the surface of the electrode material is formed through the effect of electrostatic force [8]. Pseudocapacitors store energy through rapid and reversible redox reactions or intercalation in the electrode material [9]. Thus, the selection of electrode materials is crucial for the performance of supercapacitors.

Molybdenum disulfide (MoS_2_) is a two-dimensional material composed of molybdenum and sulfur atoms, belonging to the family of transition metal dichalcogenides [10,11]. In the structure of MoS_2_, a Mo atomic layer is sandwiched between two sulfur atomic layers, forming a two-dimensional layered structure similar to graphene [12,13,14]. This layer structure imparts unique properties to MoS_2_, such as excellent electrical, optical, and mechanical properties [15,16]. Within the interlayer space of molybdenum disulfide, electrolyte ions and molecules can diffuse, providing convenience for its electrochemical and catalytic applications [17,18,19]. The atomic-level thickness and two-dimensional structure of MoS_2_ make it an ideal choice for researching and developing advanced materials and devices [20]. Specifically, the central Mo atoms exhibit oxidation states ranging from +2 to +6, creating an interlayer space that allows for the diffusion of electrolyte ions into the interlayers for Faraday reactions [21]. These distinctive features play a critical role in enhancing charge storage capabilities, ultimately enabling MoS_2_ to achieve an impressive theoretical specific capacitance of approximately 1000 F g^−1^ [22]. The few-layered treatment of MoS_2_ is able to expose more active sites, increase contact area with the electrolyte, and shorten the ion diffusion distance to further effectively enhance its electrochemical performance [23]. However, due to the challenges in preparing few-layered MoS_2_ and the high technical requirements involved, the synthesis of few-layered MoS_2_ faces significant hurdles.

In this work, few-layered MoS_2_ is successfully prepared by the one-pot hydrothermal method with the effect of NaBH_4_. Furthermore, the influence of different NaBH_4_ dosages on few-layered MoS_2_ is studied. The presence of few-layered MoS_2_ after the NaBH_4_ treatment is obtained using scanning electron microscopy (SEM) and transmission electron microscopy (TEM). MoS_2_-0.3894 exhibits the excellent supercapacitor performance, including a wide potential window of 0.9 V and a high specific capacitance of 150 g^−1^ at 1 A g^−1^. This work provides a simple way to achieve few-layered MoS_2_, which has a significant impact on the development of two-dimensional layered materials.

## 2. Materials and Methods

### 2.1. Preparation of MoS_2_ Nanosheets

First, 0.8225 g of sodium molybdate dihydrate (Na_2_MoO_4_·2H_2_O) and 0.7370 g of thioacetamide (C_2_H_5_NS) were dissolved in deionized water (25 mL) and stirred for 30 min. After the mixture was transferred to a Teflon-lined stainless-steel autoclave (100 mL), it was heated to 200 °C and kept for 20 h. The resulting product was washed several times with deionized water and ethanol, and then the sample was dried. Finally, the dried sample was ground into powder in a mortar to obtain MoS_2_.

### 2.2. Preparation of MoS_2_ with Different Amounts of NaBH4

First, 0.2595, 0.3894 and 0.5192 g of sodium borohydride (NaBH_4_) was dissolved in homogeneous mixture composed of Na_2_MoO_4_·2H_2_O and C_2_H_5_NS, and then stirred for 30 min. The rest of the operation process was the same as for the preparation of MoS_2_ nanosheets. According to the various dosages of NaBH_4_, the product samples are named MoS_2_, MoS_2_-0.2595, MoS_2_-0.3894, and MoS_2_-0.5192.

### 2.3. Material Characterizations

XRD patterns were collected on X-ray diffraction (PANalytical Empyrean, Bruker, Germany) with Cu K radiation (λ = 0.154 nm). Raman spectra were obtained by a spectrometer (TACHI Regulus8220) with 532 nm laser excitation. Materials nanostructure characterizations come from Field emission scanning electron microscope (FE-SEM, HiTACHI Regulus8220) and Energy-dispersive X-ray spectroscopy (EDX, Oxford EDX, with INCA software, INCA V7.5), transmission electron microscope (TEM, JEM-2100) with configured EDX.

## 3. Results and Discussion

**Figure 1:**
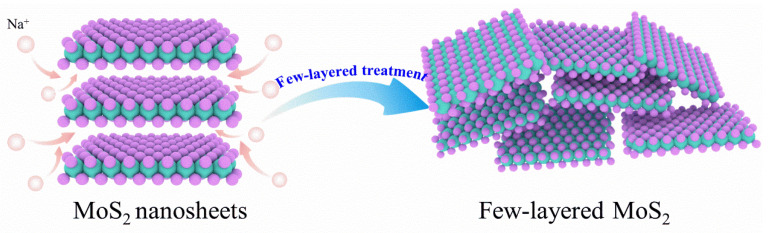
Schematic diagram of few-layered MoS_2_ preparation.

As shown in Figure 1, the layered-material MoS_2_ can be prepared into few-layered MoS_2_ with the effect of NaBH_4_. NaBH_4_ is an excellent exfoliating and reducing agent. The alkali metal Na^+^ ions have a significant exfoliating effect during synthesis processing. The group of BH_4_^−^ exhibits strong reducing properties. In addition, various NaBH_4_ have different effects on the exfoliation of MoS_2_ layers [24,25]. The products are named MoS_2_, MoS_2_-0.2595, MoS_2_-0.3894, and MoS_2_-0.5192.

**Figure 2:**
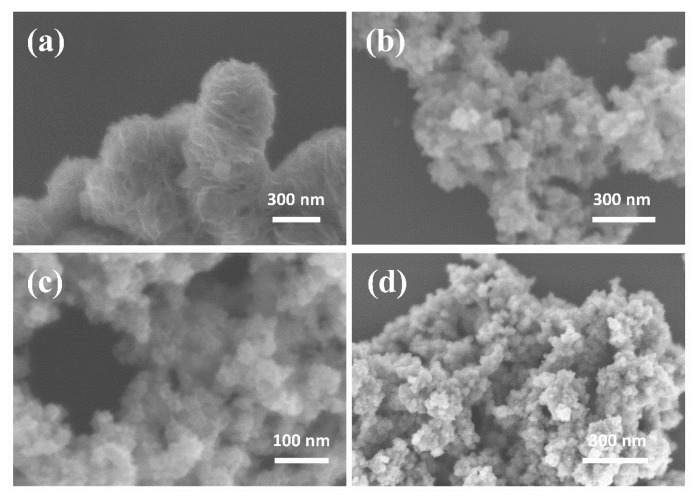
(**a**–**d**) The SEM images of MoS_2_, MoS_2_-0.2595, MoS_2_-0.3894 and MoS_2_-0.5192.

The microscopic morphologies of all the samples obtained using scanning electron microscopy (SEM) are illustrated in Figure 2. As shown in the SEM images for MoS_2_, MoS_2_-0.2595, MoS_2_-0.3894, and MoS_2_-0.5192, it is clear that the nanoflowers of MoS_2_ gradually disintegrated and agglomerated with an increase in the NaBH_4_ dosage, which reflects the significant effect of NaBH_4_ on MoS_2_ morphology.

**Figure 3:**
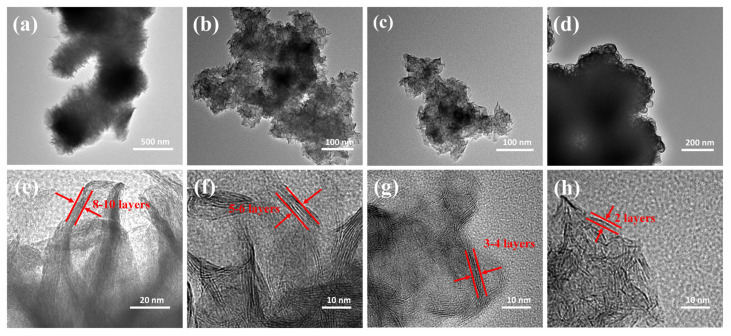
(**a**–**d**) The TEM images of MoS_2_, MoS_2_-0.2595, MoS_2_-0.3894 and MoS_2_-0.5192. (**e**–**h**) The HRTEM images of MoS_2_, MoS_2_-0.2595, MoS_2_-0.3894 and MoS_2_-0.5192.

In order to clearly demonstrate this result, the morphology of MoS_2_ treated with NaBH_4_ was also observed using transmission electron microscopy (TEM). As shown in Figure 3a, the nanoflowers of MoS_2_ are composed of a large number of nanosheets. Interestingly, for MoS_2_-0.2595 and MoS_2_-0.3894, the diameters of the nanoflowers are reduced, and their thickness is noticeably decreased. However, for MoS_2_-0.5192, the nanosheets are much stacked, which resulted from the nanosheets of MoS_2_ re-agglomerating after severe few-layered exfoliation. The layered structure of MoS_2_ was observed using high-resolution transmission electron microscopy (HRTEM), and the characteristics of the multiple layers are shown in Figure 3e. As depicted in Figure 3f–h, MoS_2_-0.2595 consists of approximately 5–6 layers, MoS_2_-0.3894 contains around 3–4 layers, and MoS_2_-0.5192 only exhibits 2 layers, indicating that NaBH_4_ significantly influences the exfoliation process. Alkali metal Na^+^ ions can intercalate into the interlayers of MoS_2_ and achieve exfoliation of the layered materials during the hydrothermal process. The TEM results indicate that the amount of NaBH_4_ is crucially related to the exfoliation effect. A significant amount of NaBH_4_ can lead to evident fragmentation of MoS_2_, ultimately resulting in its re-aggregation. The microstructure of materials plays a critical role in determining its electrochemical performance. Upon exfoliation of MoS_2_, its increased interlayer spacing allows for improved ion transport and accessibility, leading to enhanced electrochemical reactions. Furthermore, the expanded specific surface area provides more active sites for electrochemical processes, ultimately boosting the material’s performance in energy storage applications. Additionally, the presence of more defects can promote electrolyte penetration and enhance charge transfer kinetics, further optimizing the electrochemical properties of electrode materials. However, the re-aggregation of few-layered MoS_2_ can inhibit its electrochemical performance.

**Figure 4:**
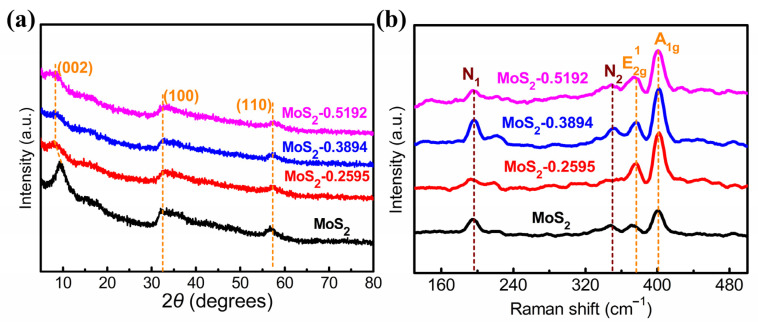
(**a**,**b**) The XRD patterns and Raman spectra of MoS_2_, MoS_2_-0.2595, MoS_2_-0.3894, and MoS_2_-0.5192.

To investigate the crystal structure of all samples, X-ray diffraction (XRD) was applied. As shown in Figure 4a, there are three characteristic peaks located at 9.3°, 32.2°, and 56.9°, which correspond to the (002), (100), and (110) planes of MoS_2_ (JCPDS no. 37-1492), respectively [26]. The interlayer spacing was about 0.94 nm after calculation using the Bragg formula [27]. The wide interlayer spacing results from the intercalation of alkali metal Na^+^ ions. On the one hand, the (002) peak of MoS_2_ shifts from 9.4° to 8.2°, indicating the exfoliation of MoS_2_ into few layers with the increase in the amount of NaBH_4_. On the other hand, the (002) peak intensity of MoS_2_ weakens after exfoliation, which resulted in defects that affected its X-ray diffraction and weaken the intensity of the characteristic peak. Large interlayer spacing and numerous defects can have a significant impact on the electrochemical performance of electrode materials. A large interlayer spacing helps to improve ion diffusion within the material, enhances electrolyte permeability, and facilitates more effective charge–discharge processes. An increased number of defects can provide additional active sites, thereby promoting ion and electron transfer, and enhancing the reactivity and electrochemical performance of the electrode [28]. Therefore, the few-layered treatment is often considered key technology for improving electrochemical performance.

Further phase analysis of MoS_2_, MoS_2_-0.2595, MoS_2_-0.3894, and MoS_2_-0.5192 was conducted using Raman spectroscopy, and the results are shown in Figure 4b, revealing characteristic peaks of 1T-MoS_2_ and 2H-MoS_2_ [29]. Two typical vibration modes of 2H-MoS_2_, E_2g_^1^ and A_1g_, can be observed at 377.0 cm^−1^ and 401.8 cm^−1^, respectively. Interestingly, the characteristic peaks corresponding to 1T exhibited blue shifts toward the shortwave region. Peaks such as j_1_, j_2_, j_3_, and E_1g_ shifted, accompanied by the appearance of two new peaks, N_1_ at 195.2 cm^−1^ and N_2_ at 352.0 cm^−1^. The blue shift phenomenon often coincides with the generation of new peaks, highlighting structural changes within the material. According to the Raman spectroscopy results, the sample contained part of 1T-MoS_2_. NaBH_4_ provided additional electrons for the formation of 1T-MoS_2_ due to its certain reduction properties during the exfoliation process of MoS_2_. 1T-MoS_2_ possesses high electrical conductivity, facilitating electron and ion transport to improve the response rate and charge–discharge efficiency of the electrode.

**Figure 5:**
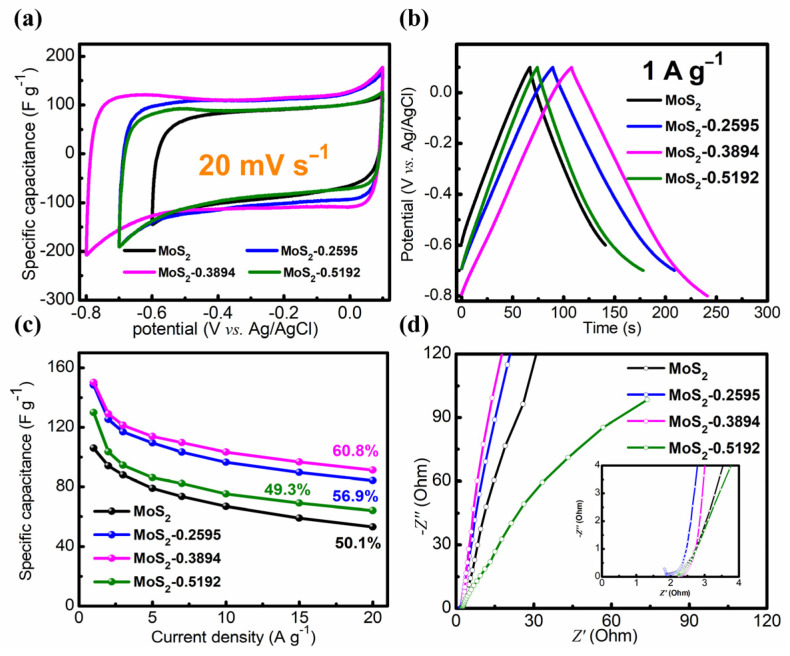
(**a**–**d**) The cyclic voltammetry (CV) curves, galvanostatic charge–discharge (GCD) curves, rate performance and electrochemical impedance spectroscopy (EIS) of MoS_2_, MoS_2_-0.2595, MoS_2_-0.3894 and MoS_2_-0.5192.

To display the supercapacitor performance of all samples, cyclic voltammetry (CV), galvanostatic charge–discharge (GCD), and electrochemical impedance spectroscopy (EIS) were evaluated using the three-electrode configuration. As shown in Figure 5a, the potential windows for MoS_2_, MoS_2_-0.2595, MoS_2_-0.3894, and MoS_2_-0.5192 are at 0.7, 0.8 and 0.9 V. Furthermore, as shown in Figure S1, the potential window for MoS_2_-0.3894 is from −0.8 to 0.1 V, which implies that MoS_2_-0.3894 has the largest potential window. Expanding the potential window not only enhances the capacitance capacity, contributing to the improved energy density and power density of supercapacitors, but also boosts response rates, enhancing the electrochemical kinetics performance of electrode materials. The expanded potential window was derived from the formation of the partial metallic phase of MoS_2_ during the exfoliation process, thereby enhancing the conductivity, and contributing to the properties of electrode materials. Additionally, the areas surrounded by the CV curves represent the capacitance of the electrode materials. It is clear that MoS_2_-0.3894 has the largest area of the CV curve compared to the other electrode materials, indicating the highest specific capacitance.

In order to more accurately reflect the specific capacitance, the GCD curves of each electrode are displayed in Figure 5b. There were some specific capacitances of 105.9, 148.2, 150 and 129.8 F g^−1^ for MoS_2_, MoS_2_-0.2595, MoS_2_-0.3894 and MoS_2_-0.5192, respectively, at a current density of 1 A g^−1^. Obviously, the specific capacitance of MoS_2_ was improved with the assistance of NaBH_4_. As shown in Figure 5c, the rate performances of MoS_2_, MoS_2_-0.2595, MoS_2_-0.3894, and MoS_2_-0.5192 are displayed as 50.1%, 56.9%, 60.8%, and 49.3%, respectively, with a current density from 1 to 20 A g^−1^. As the amount of NaBH_4_ increased, the exfoliation of few-layered MoS_2_ becomes more pronounced, leading to the formation of a larger specific surface area and an increased presence of metallic phase MoS_2_, which enhanced the rate performance. However, in the case of MoS_2_-0.5192, the re-stacking of exfoliated few-layered nanosheets resulted in a reduction in the specific surface area, impeding the diffusion of ions. As depicted in Table 1, following treatment with NaBH_4_, the voltage window of MoS_2_ was expanded, the specific capacitance of MoS_2_ was enhanced, and the rate performance of MoS_2_ was improved. These results adequately demonstrate that few-layered MoS_2_ displays outstanding electrochemical performance.

The EIS of each sample was measured, and the results are shown in Figure 5d. The EIS consists of high frequency and low frequency regions. In the high-frequency range, it typically represents the double-layer capacitance of the electrolyte at the electrode surface and the charge transfer process. In the low-frequency range, it usually represents the pseudocapacitance effect of the electrode material and the charge transfer process between the electrode and the electrolyte. MoS_2_-0.3894 had the smallest radius, indicating the lowest charge transfer resistance in the high-frequency region. Furthermore, the slope of MoS_2_-0.3894 was the biggest in the low frequency range compared to the other electrode materials, implying its capacitive-like behavior. Therefore, this indicates that MoS_2_-0.3894 has low equivalent series resistance (ESR) and high conductivity.

As shown in Figure S2, the CV and GCD curves of MoS_2_, MoS_2_-0.2595, MoS_2_-0.3894 and MoS_2_-0.5192 are shown as a series of scan rates and current densities. It is clear that the CV curves are all rectangle-like, while the GCD curves are all triangle-like, which indicates the capacitive-like behavior of all electrodes. To determine the dynamics of each electrode, the b-value fitting of each electrode is shown. The CV curves of MoS_2_-0.3894 electrode at various scan rates of 5, 10, 20, 30, 50, 70 and 100 mV s^−1^ were selected. The b-value fitting was calculated with the formula *i*(*v*) = *av^b^*, where *i* represents the current density (A g^−1^), *v* represents the scan rate (V s^−1^), *a* and *b* are both constant, respectively [30]. When the b-value is close to 0.5, it indicates semi-diffusion-controlled behavior, whereas a b-value close to 1 indicates capacitance-controlled behavior [31]. According to Figure S3, the b-value had a range from 0.75 to 1, suggesting capacitive-like behavior for the MoS_2_-0.3894 electrode. To clarify the contribution of capacitance behavior, the formula of *i*(*v*) = *k*_1_*v* + *k*_2_*v*^1/2^ was used for fitting, where *k*_1_*v* represents the contribution of capacitance behavior, while *k*_2_*v*^1/2^ represents the contribution of semi-diffusion behavior [32]. Figure S4 shows the fitting calculations of the capacitance contribution at different scan rates of 10, 20, 30, 50, 70, and 100 mV s^−1^. Moreover, the capacitance contributions increased from 77.1% to 90.3% at scan rate of 10, 20, 30, 50, 70 and 100 mV s^−1^, indicating high capacitance behavior.

## 4. Conclusions

In summary, few-layered MoS_2_ was successfully prepared using the one-pot hydrothermal method with the assistance of NaBH_4_. The exfoliation effects of different dosages of NaBH_4_ were also demonstrated in the results of the SEM and TEM images. Furthermore, a part of metallic phase MoS_2_ was obtained in the process of exfoliation. In terms of the electrochemical performance, the optimal sample of MoS_2_-0.3894 had a wide potential window of 0.9 V, the specific capacitance 150 F g^−1^ at 1 A g^−1^, and a high rate performance of 60.8%. NaBH_4_ plays an important role in the preparation of few-layered MoS_2_. This work provides a simple and effective solution for the preparation of few-layered two-dimensional materials.

## Figures and Tables

**Table 1:** The potential windows, specific capacitance and rate performance of MoS_2_, MoS_2_-0.2595, MoS_2_-0.3894, and MoS_2_-0.5192.

Samples	MoS_2_	MoS_2_-0.2595	MoS_2_-0.3894	MoS_2_-0.5192
Potential window (V)	0.7	0.8	0.9	0.8
Specific capacitance (F g^−1^)	106	148.2	150	130
Rate performance (%)	50.1	56.9	60.8	49.3

## Data Availability

Data is contained within the article.

## Supplementary Materials

The supporting information can be downloaded at: https://www.mdpi.com/article/10.3390/nano14110968/s1, Text S1: Experimental Section; Text S2: Electrochemical measurements and evaluations; Figure S1: The different potential windows of MoS_2_-0.3894 for the cyclic voltammetry (CV) and galvanostatic charge–discharge (GCD) curves at a same scan rate of 20 mV s^−1^ and current density of 1 A g^−1^; Figure S2: The CV and GCD curves of MoS_2_-0.3894 at a series of scan rates and current densities; Figure S3: b-value for MoS_2_-0.3894 electrode; Figure S4: CV partition analysis showing the capacitive contribution to the total current at select scan rates of 10, 20, 30, 50, 70 and 100 mV s^−1^; Figure S5: Normalized contribution ratio of capacitive capacitance at different scan rates.
