# Supplementary material for: Preparation of Few-Layered MoS2 by One-Pot Hydrothermal Method for High Supercapacitor Performance
Source: Nanomaterials (Basel). 2024 Jun 2;14(11):968. doi: 10.3390/nano14110968 (PMC11173613; doi:10.3390/nano14110968)
Supplement: Supplementary file 1 [file nanomaterials-14-00968-s001.zip › nanomaterials-3008160-supplementary.pdf]

Supporting information for

Article

Preparation of Few-Layered MoS_2 by One-Pot Hydrothermal Method for High Supercapacitor Performance

Qingling Jia [1], Qi Wang [1], Lingshuai Meng [1], Yujie Zhao [1], Jing Xu [1], Meng Sun [1], Zijian Li [1], Han Li [1,*], Huiyu Chen [2,*] and Yongxing Zhang [1]

[1] Anhui Province Key Laboratory of Pollutant Sensitive Materials and Environmental Remediation, Anhui Province Key Laboratory of Intelligent Computing and Applications, Anhui Province Industrial Generic Technology Research Center for Alumics Materials, Huaibei Normal University, Huaibei 235000, China; zyx07157@mail.ustc.edu.cn (Y.Z.)

[2] School of Materials Science and Engineering, North University of China, Taiyuan 030051, China

* Correspondence: lh2017@mail.ustc.edu.cn (H.L.); hychen@nuc.edu.cn (H.C.)

Text S1: Experimental Section

Chemicals: Sodium molybdate dihydrate ($Na_2MoO_4 \cdot 2H_2O$, 99%), thioacetamide (C_2H_5NS, 99%), sodium borohydride ($NaBH_4$, 98%), anhydrous sodium sulfatewere (Na_2SO_4, 99%), potassium chloride (KCl, 99.8%) and ethanol (C_2H_6O, 95%) were purchased from Sinopharm Chemical Reagent Co. Ltd. (Shanghai, China). All chemicals were used without any further purification.

Text S2: Electrochemical measurements and evaluations

This experiment uses a three electrode testing method to test the electrochemical performance of the sample on an electrochemical workstation. The three electrodes refer to the working electrode (active materials: carbon black: PVDF=8:1:1), reference electrode (Ag/AgCl electrode), and counter electrode(Pt electrode), respectively. Among them, the electrolytic cell solution in the three electrode testing system is 1 M Na_2SO_4 solution.

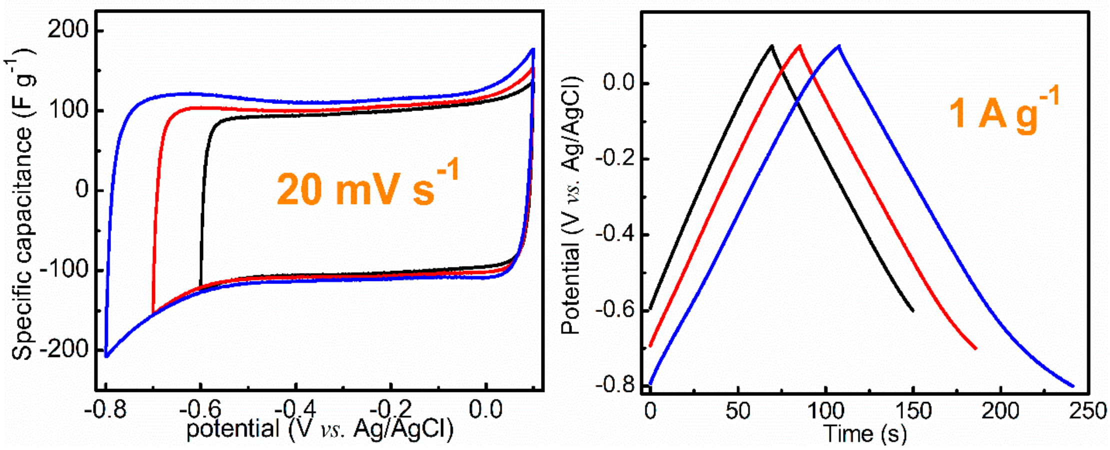

Figure S1. The different potential windows of MoS_2-0.3894 for the cyclic voltammetry (CV) and galvanostatic charge-discharge (GCD) curves at a same scan rate of 20 mV s^{-1} and current density of 1 A g^{-1}.

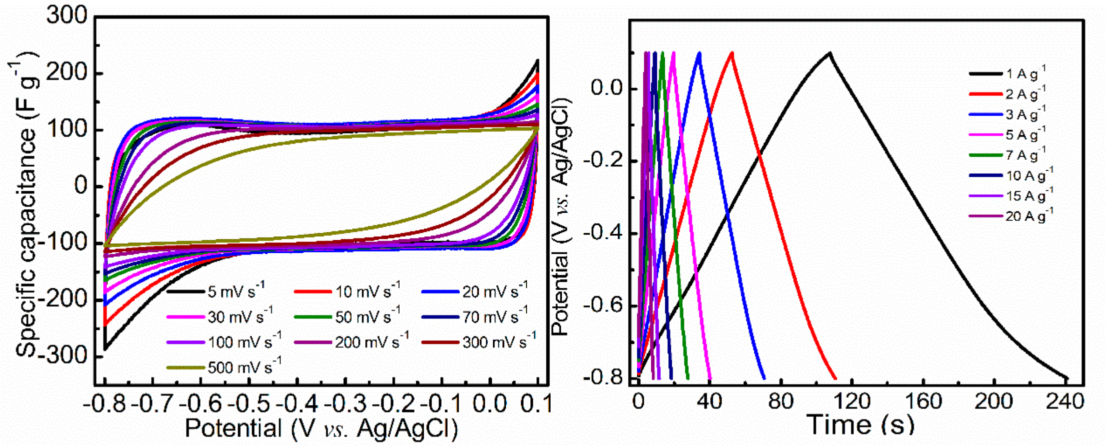

Figure S2. The CV and GCD curves of MoS_2-0.3894 at a series of scan rates and current densities.

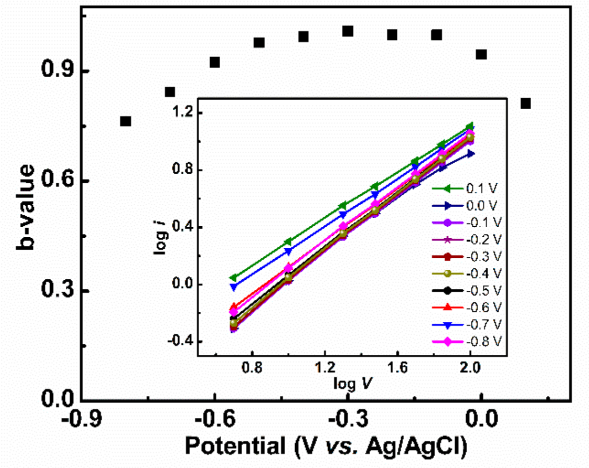

Figure S3. *b*-value for MoS_2-0.3894 electrode.

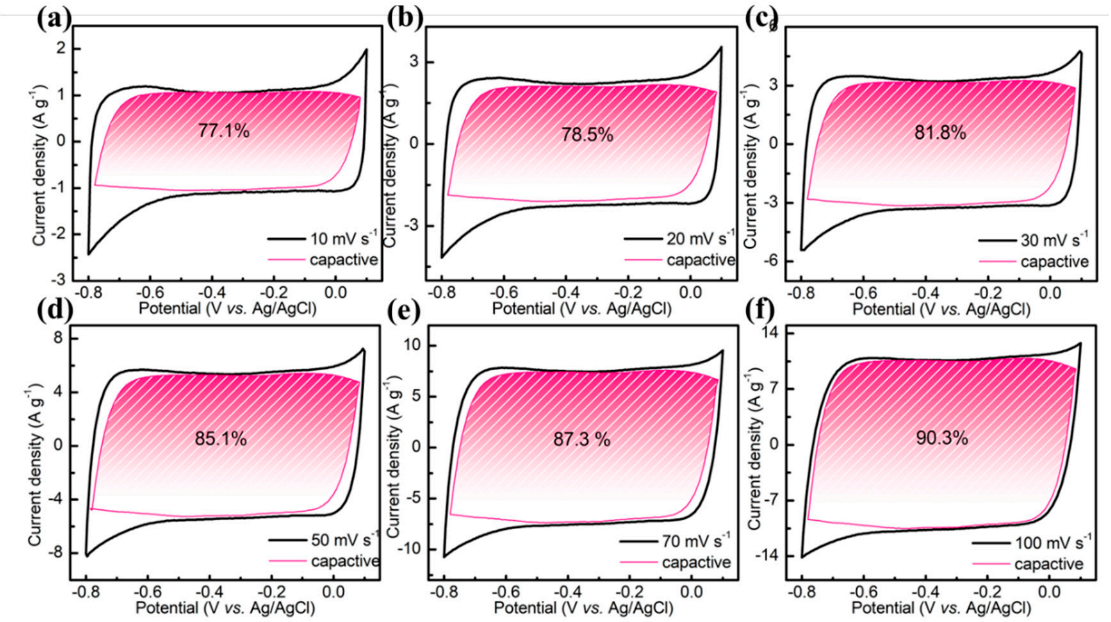

Figure S4. (a-f) CV partition analysis showing the capacitive contribution to the total current at select scan rates of 10, 20, 30, 50, 70 and 100 mV s^{-1}.

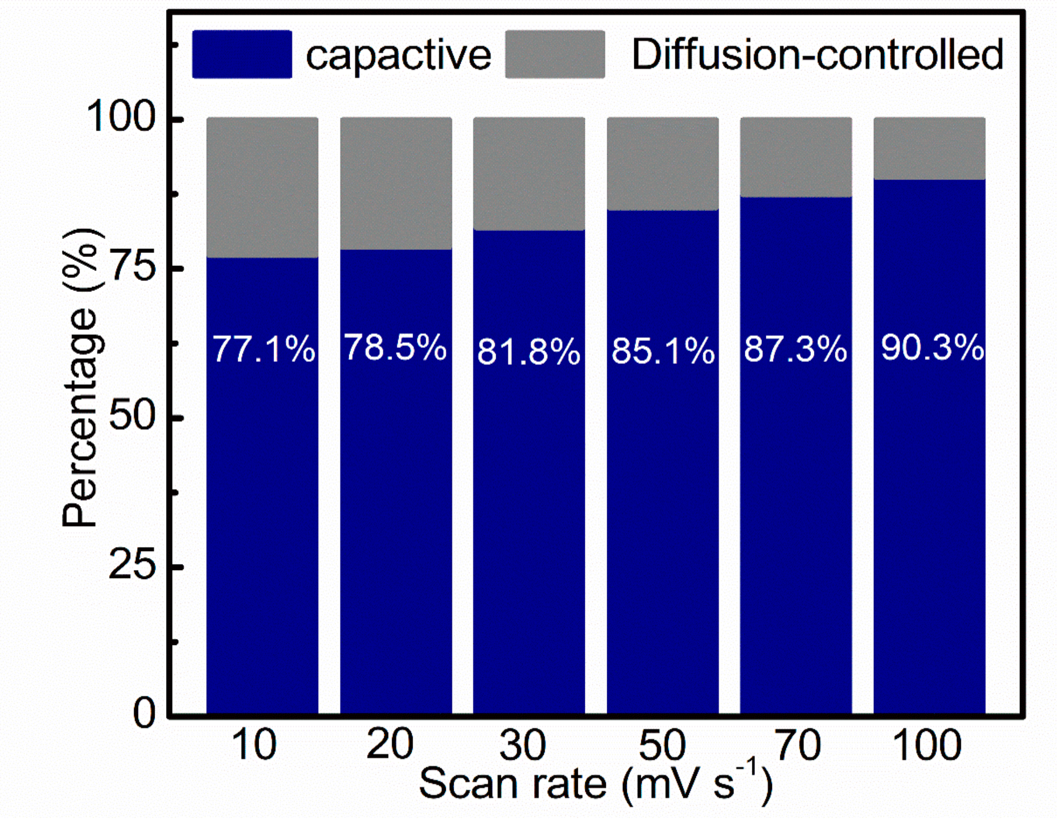

Figure S5. Normalized contribution ratio of capacitive capacitance at different scan rates.
